# Negative impact of leukoaraiosis on the incidence of brain metastases in patients with lung cancer

**DOI:** 10.1007/s11060-017-2574-x

**Published:** 2017-07-27

**Authors:** Nakamasa Hayashi, Koichi Mitsuya, Yoko Nakasu, Tateaki Naito, Fumiharu Ohka, Toshiaki Takahashi

**Affiliations:** 1 Division of Neurosurgery, Shizuoka Cancer Center Hospital, Nagaizumi, Shizuoka, 411-8777 Japan; 2 Division of Thoracic Oncology, Shizuoka Cancer Center Hospital, Shizuoka, 411-8777 Japan; 3 Department of Neurosurgery, Nagoya University, Nagoya, 466-8550 Japan

**Keywords:** Brain metastasis, Leukoaraiosis, Lung cancer, Magnetic resonance imaging, White matter lesion

## Abstract

The embolization of cancer cells to cerebral vessels occurs early in the multi-step metastatic process. We aimed to determine whether the presence of leukoaraiosis (LA) before treatment would predict the development of brain metastases (BM) in patients with lung cancer. Between January 2014 and June 2015, 1007 patients underwent initial (i.e., prior to any chemotherapy) or routine magnetic resonance (MR) imaging of the brain and exhibited no evidence of BM. Of these, 189 underwent repeat MR imaging; 34 of 189 patients (18%) developed new BM, whereas 155 patients did not. LA was retrospectively evaluated according to Fazekas scale on the initial screening MR images of these 189 patients. The frequency of grade 0 periventricular hyperintensity (PVH) was greater among patients with BM, compared to those without BM (p = 0.001). In a multivariate analysis, patients with adenocarcinoma (95% confidence interval [CI] 1.8–171.8) and small cell carcinoma (95% CI 1.4–172.4) respectively developed BM at 9.3- and 8.8-fold higher rates than those with squamous cell carcinoma. Patients with grade 0 PVH developed BM at a rate 3.5-, 8.6-, and 3.6-fold higher rates than those with grade 1 (95% CI 1.4–9.0), 2 (95% CI 2.4–41.9), and 3 (95% CI 1.02–15.0), respectively. Lung cancer patients with grade 0 PVH on initial MR images have a high subsequent incidence of BM. PVH is a useful method for evaluating risk of BM.

## Introduction

Overall, 10–15% of patients with lung cancer have brain metastases (BM) at diagnosis, and an additional 20–25% will develop BM during the course of their illness [1]. The multi-step metastatic process involves several biological mechanisms, including embolization, survival in circulation, endothelial invasion, angiogenesis, extravasation, proliferation within the brain parenchyma, and resistance to glial immune surveillance [2].

Leukoaraiosis (LA) is an age-related type of cerebral white matter degenerations in the centrum semiovale, and is characterized by hyperintensity on T2-weighted magnetic resonance (MR) images [3, 4]. Moody et al. demonstrated significantly lower afferent microvascular density in LA lesions within deep white matter, compared to the corresponding deep white matter in a healthy subject, indicating that LA is a generalized cerebrovascular disease process [5]. Most pathogenetic schemes attribute LA to an insufficient blood supply to the cerebral deep white matter. LA findings on MR images have been reported to associate with high risks of stroke, dementia, and depression [6, 7].

We hypothesized that small vessel ischemic disease might play a preventive role against BM by reducing the frequency of cancer cell embolization to cerebral vessels and inhibiting proliferation within the brain parenchyma consequent to poor blood supply and nutrition. The aim of this longitudinal study was to determine the relationship between the grades of LA on initial MR images of lung cancer patients and the occurrence of BM.

## Patients and methods

Between January 2014 and June 2015, 1007 patients with lung cancer underwent initial (i.e., prior to any chemotherapy) or routine MR imaging of the brain and were found to exhibit no evidence of BM. Of these patients, 189 of 1007 patients underwent repeat MR imaging in the same period. Thirty-four of the 189 patients (18%) developed new BM, whereas 155 patients did not. In the 189 patients, white matter changes were retrospectively rated on fluid attenuated inversion recovery (FLAIR) images from initial brain MR screenings performed at Shizuoka Cancer Center Hospital.

MR imaging was performed on a 1.5-T MR imaging system (Intera; Philips Healthcare, Best, Netherlands). The following imaging parameters were used: repetition time/echo time, 11,000/100 ms; number of excitations for FLAIR imaging, 2.

**Fig. 1:**
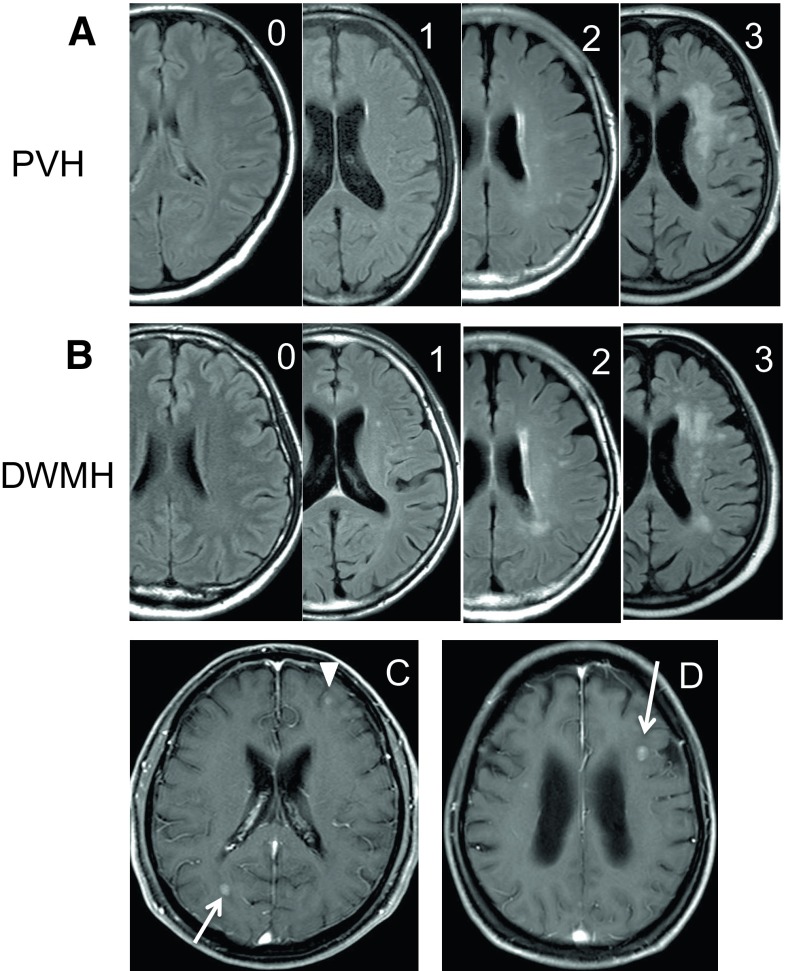
Representative magnetic resonance (MR) images. **a** Fluid attenuated inversion recovery images showing the grade of periventricular hyperintensity (PVH) according to the Fazekas scale. **b** Showing deep white matter hyperintense signals (DWMH). The *number in the upper right corner* indicates the grade. **c** Gadolinium-enhanced T1-weighted images showing the appearance of brain metastases in the vascular border zones. Note that two lesions are located in the anterior border zone situated between the anterior cerebral artery and middle cerebral artery supply territories (*arrowhead*), and in the posterior border zone situated between the anterior cerebral artery and posterior cerebral artery supply territories (*arrow*). **d** A lesion is located in the subcortical border zone situated in the most distal vascular field (*arrow*)

LA was graded according to the Fazekas scale [8], which is used to describe the different types of hyperintense signal abnormalities surrounding the ventricles and in the deep white matter. Periventricular hyperintensity (PVH) was graded as 0, absent; 1, cap or pencil-thin lining; 2, smooth halo; 3, irregular and extending into the deep white matter (Fig. 1a). Deep white matter hyperintense signals (DWMH) were rated as 0, absent; 1, punctate foci; 2, little focal confluence; 3, large confluent areas (Fig. 1b). The ratings were performed by three neurosurgeons (KM, FO, YN) who were blinded to the patient’s BM statuses.

The collected clinical data included sex, date of birth, histological type of lung cancer, epidermal growth factor receptor status, type of treatment for lung cancer, general complications associated with LA, use of antiplatelet drugs, smoking status, and dates of screening and the last MR imaging or the imaging session during which BM was detected.

Approval for this study was obtained from the institutional research ethics board of Shizuoka Cancer Center (number: 27-J156-27-1-3). The requirement for individual written informed consent was waived because this retrospective study was based on record extracted from a database.

Clinico-pathological characteristics were compared between patients with BM and without BM using the Chi square test and Wilcoxon test. The Chi square test and Wilcoxon test were used to examine the association between Fazekas grade and clinical characteristics. Distributions of PVH and DWMH grades were compared between patients with BM and without BM using the Chi square test. A multivariate analysis via logistic regression was performed to assess risk factors for the occurrence of BM. BM-free survival was calculated from the date of the initial screening MR imaging to that of the BM diagnosis, using Kaplan–Meier estimates. A p-value of <0.05 was considered to indicate a statistically significant difference. Analyses were performed using JMP^®^ software (Version 11, SAS institute Inc., Tokyo, Japan).

## Results

### Patient characteristics

The clinical characteristics of this study population are listed in Table 1. The median age at the time of initial MR imaging was 66 years among patients with BM and without BM. The stage of lung cancer was not statistically different between patients with BM and without BM. The most common type of tumor histology was adenocarcinoma, accounting for 73.5% of patients with BM and 56.8% of those without BM. Chemotherapy for the lung cancer was provided in half of the patients with both groups after the initial screening. Patients with and without BM did not differ significantly regarding baseline characteristics, with the exception of tumor histology.

**Table 1:** Clinical characteristics of study population at initial screening MR imaging

	Brain metastases		p-Value
	Yes (n = 34)	No (n = 155)	
Male sex	23 (67.7%)	101 (65.2%)	0.78
Median age (range)	66 (46–85)	66 (29–85)	0.74*
<65	15 (44%)	58 (37%)	0.47
≥65	19 (56%)	97 (63%)	
Stage			0.06
I	2 (5.9%)	28 (18.1%)	
II	2 (5.9%)	23 (14.8%)	
III	12 (35.3%)	48 (31.0%)	
IV	18 (52.9%)	56 (36.1%)	
Histology			0.015
Adenocarcinoma	25 (73.5%)	88 (56.8%)	
Small cell carcinoma	7 (20.6%)	25 (16.1%)	
Squamous cell carcinoma	1 (2.9%)	37 (23.9%)	
Others	1 (2.9%)	5 (3.2%)	
EGFR mutation status			0.09
Yes	5	29	
No	20	48	
Chemotherapy			0.18
Yes	19 (56%)	67 (43%)	
No	15 (44%)	88 (57%)	
Complication			
Stroke	1 (2.9%)	12 (7.7%)	0.27
Ischemic heart disease	1 (2.9%)	15 (9.7%)	0.15
Hypertension	11 (32.4%)	59 (38.1%)	0.53
Diabetes mellitus	2 (5.9%)	21 (13.6%)	0.18
Hyperlipidemia	4 (11.8%)	22 (14.2%)	0.70
Use of antiplatelet drug	2 (5.8%)	18 (11.8%)	0.28
Tabacco use	24 (70.6%)	122 (78.7%)	0.32
Median time to the last evaluation from initial screening	19 (3–96)	19 (1–120)	0.44*

*EGFR* epidermal growth factor receptor

*Wilcoxon test

The median interval between the initial screening and the last evaluation was 19 months in both groups. The intervals between the initial screening and the repeat study ranged from 1 to 12 months. Asymptomatic BM were found in 26 of 34 patients during routine follow-up MR imaging.

### Grades of LA

The clinical characteristics, including risk factors for ischemic brain disease, are listed by Fazekas grade in Table 2. PVH grades were found to correlate statistically with age, history of stroke, hypertension, and use of antiplatelet drugs. DWMH grades were statistically associated with age, histology of lung cancer, history of stroke and ischemic heart disease, hypertension, diabetes mellitus, and use of antiplatelet drugs.

**Table 2:** The relation between Fazekas grade and clinical characteristics

	PVH				p-Value	DSWH				p-Value
	0	1	2	3		0	1	2	3	
Total	31	86	42	30		37	63	50	39	
Male sex	21 (67.7%)	56 (65.1%)	26 (61.9%)	21 (70.0%)	0.90	25 (67.6%)	44 (69.8%)	29 (58.0%)	26 (66.7%)	0.61
Median age (range)	58.8 (29–75)	64.8 (43–82)	68.7 (45–79)	75.3 (57–85)	<0.0001*	59.9 (29–75)	63.8 (42–82)	68.2 (51–85)	74.1 (57–85)	<0.0001*
Histology					0.15					0.0299
Adenocarcinoma	21 (18.6%)	55 (48.7%)	25 (22.1%)	12 (10.6%)		25 (22.1%)	44 (38.9%)	28 (24.8%)	16 (14.2%)	
Small cell carcinoma	6 (18.8%)	13 (40.6%)	5 (15.6%)	8 (25.0%)		7 (21.9%)	10 (31.3%)	7 (21.9%)	8 (25.0%)	
Squamous cell carcinoma	3 (7.9%)	16 (42.1%)	9 (23.7%)	10 (26.3%)		4 (10.5%)	9 (23.7%)	11 (29.0%)	14 (36.8%)	
Others	1 (16.7%)	2 (33.3%)	3 (50.0%)	0		1 (16.7%)	0	4 (66.7%)	1 (16.7%)	
EGFR mutation status					0.89					0.80
Yes	6 (17.7%)	16 (47.1%)	8 (23.5%)	4 (11.8%)		8 (23.5%)	12 (35.3%)	9 (26.5%)	5 (14.7%)	
No	14 (20.6%)	35 (51.5%)	12 (17.7%)	7 (10.3%)		13 (19.1%)	31 (45.9%)	15 (22.1%)	9 (13.2%)	
Complication										
Stroke					0.0018					0.0027
Yes	0	2 (15.4%)	5 (38.5%)	6 (46.2%)		0	3 (23.1%)	2 (15.4%)	8 (61.5%)	
No	31 (17.6%)	84 (47.7%)	37 (21.0%)	24 (13.6%)		37 (21.0%)	60 (34.1%)	48 (27.3%)	31 (17.6%)	
Ischemic heart disease					0.18					0.0335
Yes	1 (6.3%)	5 (31.3%)	5 (31.3%)	5 (21.3%)		1 (6.3%)	2 (12.5%)	7 (43.8%	6 (37.5%)	
No	30 (17.3%)	81 (46.8%)	37 (21.4%)	25 (14.5%)		36 (20.8%)	61 (35.3%)	43 (24.9%)	33 (19.1%)	
Hypertension										0.0023
Yes	7 (10.0%)	28 (40.0%)	15 (21.4%)	20 (28.6%)	0.0022	8 (11.4%)	20 (28.6%)	18 (25.7%)	24 (34.3%)	
No	24 (20.2%)	58 (48.7%)	27 (22.7%)	10 (8.4%)		29 (24.4%)	43 (36.1%)	32 (26.9%)	15 (12.6%)	
Diabetes mellitus										0.0280
Yes	1 (4.4%)	11 (47.8%)	5 (21.7%)	6 (26.1%)	0.20	1 (4.4%)	5 (21.7%)	10 (43.5%)	7 (30.4%)	
No	30 (18.1%)	75 (45.2%)	37 (22.3%)	24 (14.5%)		36 (21.7%)	58 (34.9%)	40 (24.1%)	32 (19.3%)	
Hyperlipidemia					0.79					0.26
Yes	3 (11.5%)	11 (42.3%)	7 (26.9%)	5 (19.2%)		2 (7.7%)	8 (30.8%)	9 (34.6%)	7 (26.9%)	
No	28 (17.2%)	75 (46.0%)	35 (21.5%)	25 (15.3%)		35 (21.5%)	55 (33.7%)	41 (25.2%)	32 (19.6%)	
Use of antiplatelet drug					0.0008					<0.0001
Yes	0	5 (25.0%)	7 (35.0%)	8 (40.0%)		0	2 (10.0%)	8 (40.0%)	10 (50.0%)	
No	31 (18.6%)	79 (47.3%)	35 (21.0%)	22 (13.7%)		37 (22.2%)	60 (35.9%)	41 (24.6%)	29 (17.4%)	
Tobacco use					0.42					0.45
Yes	25 (17.1%)	65 (44.5%)	30 (20.6%)	26 (17.8%)		30 (20.6%)	47 (32.2%)	36 (24.7%)	33 (22.6%)	
No	6 (14.0%)	21 (48.8%)	12 (27.9%)	4 (9.3%)		7 (16.3%)	16 (37.2%)	14 (32.6%)	6 (14.0%)	
Number of brain metastasis					0.75					0.69
1	6	5	2	2		5	3	4	3	
2–4	3	3	1	1		3	3	1	1	
≥5	3	5	0	1		2	5	2	0	

*PVH* periventricular hyper-intensity, *DSWH* deep white matter hyperintense signals

*Wilcoxon test

A total of 122 lesions were observed in 32 patients. Uncountable, disseminated lesions were found in two patients, whereas solitary metastases were observed in 15 patients. Lesions were more frequently located in the cerebellum (28 lesions) and the parietal lobe (23 lesions). Sixty-two of the 122 lesions (51%) were located in the vascular border zone (Fig. 1c, d). There were no statistical differences between the number of BM and grade of LA (Table 2).

### Risk factors for the occurrence of brain metastases

**Fig. 2:**
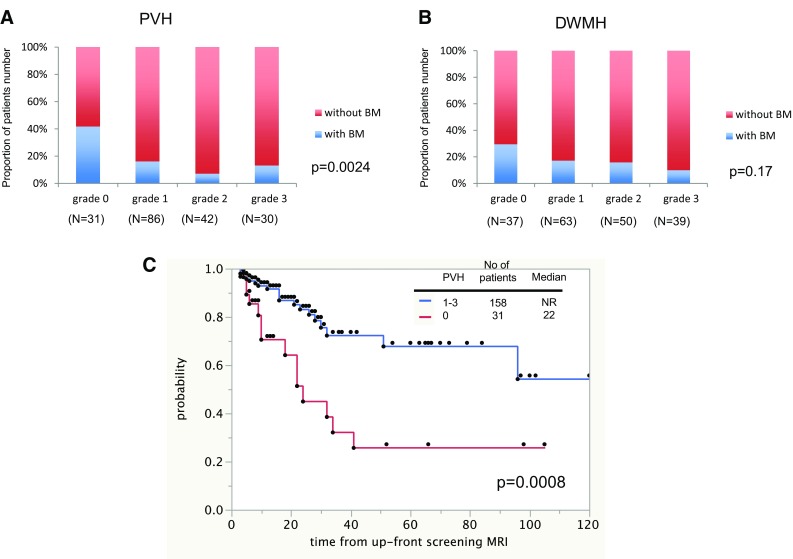
Influence of leukoaraiosis grade on the incidence of brain metastases. **a** Distributions of the grades of periventricular hyperintensity and **b** deep white matter hyperintense signals. **c** Kaplan–Meier analysis of brain-metastases-free survival according to periventricular hyperintensity grade

The frequency of grade 0 PVH was higher among patients with BM than those without BM (Fig. 2a). The distributions of PVH grades differed significantly between groups. In contrast, although the DWMH grades were similarly distributed, these differences were not statistically significant (Fig. 2b).

The lung cancer histology and PVH grade were significant risk factors for the occurrence of BM in a univariate analysis. In a multivariate analysis that incorporated the significant factors from the univariate analysis, patients with adenocarcinoma (95% confidence interval [CI] 1.8–171.8) and small cell carcinoma (95% CI 1.4–172.4) were found to develop BM at 9.3- and 8.8-fold higher rates, respectively, than those with squamous cell carcinoma. Patients with grade 0 PVH developed BM at 3.5-, 8.6-, and 3.6-fold higher rates than those with grade 1 (95% CI 1.4–9.0), 2 (95% CI 2.4–41.9), and 3 PVH (95% CI 1.02–15.0), respectively (Table 3).

**Table 3:** Multivariate analysis of risk factors for occurrence of brain metastases

	Odds ratio	95% CI	p-Value
Histology			
Adenocarcinoma vs squamous cell carcinoma	9.34	1.80–171.77	0.0043
Small cell carcinoma vs squamous cell carcinoma	8.82	1.39–172.44	0.0182
Adenocarcinoma vs small cell carcinoma	1.06	0.40–3.05	0.91
PVH grade			
0 vs 1	3.48	1.37–9.00	0.0091
0 vs 2	8.62	2.37–41.87	0.0007
0 vs 3	3.60	1.02–15.04	0.0463
1 vs 2	2.48	0.74–11.37	0.15
1 vs 3	1.03	0.31–4.05	0.96
2 vs 3	0.42	0.07–2.14	0.29

### Brain-metastases-free survival

BM occurred in 13 of 31 patients (41.9%) with grade 0 PVH, and in 21 of 158 patients (13.3%) with grade 1–3 PVH. The median BM-free survival for patients with grade 0 PVH was 22 months (95% CI, 10-unavailable), whereas a median value was not reached for patients with grade 1–3 PVH (95% CI, 96-unavailable) (Fig. 2c). The median BM-free survival for patients with grade 0 PVH was significantly shorter than that for patients with grade 1–3 PVH (p = 0.0008).

## Discussion

The results of our present study demonstrate that lung cancer patients without LA on initial MR images have a higher likelihood of BM development, compared to those with LA. We rated LA according to the Fazekas scale [8], a very simple, easy, and suitable clinical tool. LA is usually subdivided according to the anatomical positions of white matter lesions as immediately adjacent to the ventricle (PVH) or within the subcortical or deep white matter (DWMH). In the present study, we observed a strong correlation of PVH rather than DWMH, with the occurrence of BM in lung cancer patients.

PVH and DWMH differ with respect to pathogenesis and clinical significance. PVH is thought to result from ischemia in an arterial border-zone, possibly due to hypoperfusion caused by large vessel disease [9]. PVH has also been associated with diminished cerebral vasomotor reactivity and subsequent cerebral hypoperfusion [10], whereas DWMH is generally associated with microangiopathy [11]. Gebeily et al. reported a linear correlation of PVH with vascular risk factors such as aging and hypertension [12]. The Rotterdam Scan Study revealed that elderly people with white matter lesions had a strongly increased risk of stroke [7]. However, unlike PVH, DWMH did not exhibit a linear association with the risk of stroke [7]. In other studies, extensive PVH, but not DWMH, was independently associated with ischemic stroke, 90-day recurrent stroke, and functional stroke outcomes [9, 13, 14].

The advent of image registration and 3-dimensional structure deformation algorithms has led to increasing objective analyzes of spatial BM distribution. Bender et al. reported an increased probability of cerebellar metastases of lung cancer, compared to predictions based solely on brain volume [15]. Another study found that metastatic lesions were more likely to occur in the parieto-occipital lobes and cerebellum of non-small-cell lung cancer patients [16]. Takano et al. recently reported that the posterior fossa, anatomic watershed areas, and the gray–white matter junction were more commonly affected by BM of lung cancer [17]. Another report suggested that metastatic emboli tend to lodge in areas characterized by a sudden reduction in vascular caliber (e.g., gray–white matter junction) and in the most distal vascular field (vascular border zone) [18]. Therefore, the mechanical trapping of tumor emboli is an essential contributor to the occurrence of BM.

In the present study, we demonstrated that the LA grade was significantly associated with the incidence of BM of lung cancer. Patients with grade 0 PVH on initial MR images had a greater risk of BM, compared to those with grade 1–3 PVH. We revealed that the median BM-free survival for patients with grade 0 PVH was significantly shorter than that for patients with grade 1–3 PVH. One possible explanation of this finding is that in patients with LA, fewer tumor emboli may reach the cerebral vessels, and the poorer blood supply and nutrition might not support tumor cell proliferation within the brain parenchyma. Patients with grade 0 PVH on initial MR images should be closely followed by serial MR imaging.

Several reports have described a negative correlation between white matter lesions and BM. Mazzone et al. reported that small vessel ischemic disease protected against BM development, following a review of MR images of lung cancer patients obtained during staging to evaluate the presence of BM and features of LA [19]. Quattrocchi et al. reported a lower volume of BM at the MR imaging diagnosis among patients with brain white matter hyperintensities determined using a quantitative imaging approach [20]. That report demonstrated an inverse relationship between the volumes of white matter hyperintensities and of BM. Recently, white matter lesions were reported to associate with lower numbers of BM in an evaluation of MR images obtained for LA grading at the time of the diagnosis of BM [21]. The present study included patients with lung cancer who underwent a brain MR imaging as part of the initial or routine screening and were not diagnosed with evidence of BM. As LA was evaluated on initial brain MR images, our study truly investigated the relationship of LA grade before treatment with the subsequent incidence of BM during the course of lung cancer.

The negative impact of LA on BM might affect the treatment of BM. Prophylactic cranial irradiation has been proposed for patients with small cell lung cancer and non-small-cell lung cancer, given the life-threatening symptoms and serious quality of life impairment associated with BM [22]. However, our findings suggest that prophylactic cranial irradiation could be omitted in patients with higher grades of LA. Furthermore, stereotactic radiosurgery, rather than whole-brain radiation therapy, might be recommended for patients with small numbers of BM and higher grades of LA because of the lower incidence of new BM development. The negative impact of LA on BM might also influence decisions regarding chemotherapy, as physicians are not concerned with the blood–brain barrier and do not fear the development of new BM during systemic chemotherapy for lung cancer. Future prospective studies of BM from lung cancer should consider the LA grade.

The present study has limitations that are inherent to the retrospective design. Repeat MR imaging was usually performed at the time of systemic deterioration or during a regular follow-up evaluation. The range of interval between initial screening and repeat studies (1–12 months) might have influenced the results. Furthermore, because our study only assessed patients with lung cancer, we cannot apply our findings to other malignancies known to metastasize to the brain.

## Conclusions

Lung cancer patients with grade 0 PVH on initial MR images have a high subsequent incidence of BM. PVH is a useful method for evaluating risk of BM.

## References

[1] Mazzone P, Chao ST, Suh J, Biller J (2007). Lung cancer. Neurology of internal medicine.

[2] Luzzi KJ, MacDonald IC, Schmidt EE (1998). Multistep nature of metastatic inefficiency: dormancy of solitary cells after successful extravasation and limited survival of early micrometastases. Am J Pathol.

[3] Hachinski VC, Potter P, Merskey H (1986). Leukoaraiosis: an ancient term for a new problem. Can J Neurol Sci.

[4] Hachinski VC, Potter P, Merskey H (1987). Leuko-araiosis. Arch Neurol.

[5] Moody DM, Thore CR, Anstrom JA (2004). Quantification of afferent vessels shows reduced brain vascular density in subjects with leukoaraiosis. Radiology.

[6] O’Brien JT, Erkinjunttu T, Reisberg B (2003). Vascular cognitive impairment. Lancet Neurol.

[7] Vermeer SE, Hollander M, van Dijk EJ (2003). Silent brain infarcts and white matter lesions increase stroke risk in the general population: the Rotterdam scan study. Stroke.

[8] Fazekas F, Chawluk JB, Alavi A (1987). MR signal abnormalities at 1.5 T in Alzheimer’s dementia and normal age. AJR Am J Roentgenol.

[9] Gerdes VE, Kwa VI, ten Cate H (2006). Cerebral white matter lesions predict both ischemic strokes and myocardial infarctions in patients with established Atherosclerotic disease. Atherosclerosis.

[10] Bakker SL, de Leeuw FE, de Groot JC (1999). Cerebral vasomotor reactivity and cerebral white mater lesions in the elderly. Neurology.

[11] Fazekas F, Kleinert R, Offenbacher H (1993). Pathogenic correlates of incidental MRI white matter signal hyperintensities. Neurology.

[12] Gebeily S, Fares Y, Kordahi M (2014). Cerebral white matter hyperintensities (WMH): an analysis of cerebrovascular risk factors in Lebanon. Int J Neurosci.

[13] Kim GM, Park KY, Avery R (2014). Extensive leukoaraiosis is associated with high early risk of recurrence after ischemic stroke. Stroke.

[14] Liou LM, Chen CF, Guo YC (2010). Cerebral white matter hyperintensities predict functional stroke outcome. Cerebrovasc Dis.

[15] Bender ET, Tome WA (2011). Distribution of brain metastases: implications for non-uniform dose prescription. Br J Radiol.

[16] Quattrocchi CC, Errante Y, Gaudino C (2012). Spatial brain distribution of intra-axial metastatic lesions in breast and lung cancer patients. J Neurooncol.

[17] Takano K, Kinoshita M, Takagaki M (2016). Different spatial distribution of brain metastases from lung cancer by histological subtype and mutation status of epidermal growth factor receptor. Neuro Oncol.

[18] Hwang TL, Close TP, Grego JM (1996). Predilection of brain metastasis in gray and white matter junction and vascular border zones. Cancer.

[19] Mazzone PJ, Marchi N, Fazio V (2009). Small vessel ischemic disease of the brain and brain metastases in lung cancer patients. PLoS ONE.

[20] Quattrocchi CC, Errante Y, Mallio CA (2013). Brain metastatic volume and white matter lesions in advanced cancer patients. J Neurooncol.

[21] Berk BA, Nagel S, Hering K (2016). White matter lesions reduce number of brain metastases in different cancer: high-resolution MRI study. J Neurooncol.

[22] Pechoux CL, Sun A, Slotman BJ (2016). Prophylactic cranial irradiation for patients with lung cancer. Lancet Oncol.

